# Effect of the new silicon-based agent on the symptoms of interstitial pneumonitis

**DOI:** 10.1038/s41598-023-32745-8

**Published:** 2023-04-07

**Authors:** Masato Shimada, Yoshihisa Koyama, Yuki Kobayashi, Hikaru Kobayashi, Shoichi Shimada

**Affiliations:** 1 Department of Neuroscience and Cell Biology, Osaka University Graduate School of Medicine, 2-2 Yamadaoka, Suita, Osaka 565-0871 Japan; 2 Addiction Research Unit, Osaka Psychiatric Research Center, Osaka Psychiatric Medical Center, Osaka, 541-8567 Japan; 3 SANKEN, Osaka University, Osaka, 567-0047 Japan

**Keywords:** Inflammation, Experimental models of disease

## Abstract

Interstitial pneumonia (IP) is a collective term for diseases whose main lesion is fibrosis of the pulmonary interstitium, and the prognosis associated with acute exacerbation of these conditions is often poor. Therapeutic agents are limited to steroids, immunosuppressants, and antifibrotic drugs, which and have many side effects; therefore, the development of new therapeutic agents is required. Because oxidative stress contributes to lung fibrosis in IP, optimal antioxidants may be effective for the treatment of IP. Silicon (Si)-based agents, when administered orally, can continuously generate a large amount of antioxidant hydrogen in the intestinal tract. In this study, we investigated the effect of our Si-based agent on methotrexate-induced IP, using the IP mouse models. Pathological analysis revealed that interstitial hypertrophy was more significantly alleviated in the Si-based agent-treated group than in the untreated group (decreased by about 22%; P < 0.01). Moreover, additional morphological analysis demonstrated that infiltration of immune cells and fibrosis in the lungs were significantly inhibited by treatment with the Si-based agent. Furthermore, Si-based agent reduced oxidative stress associated with IP by increasing blood antioxidant activity. (increased by about 43%; P < 0.001). Taken together, these results suggest that Si-based agents can be effective therapeutic agents for IP.

## Introduction

Interstitial pneumonia (IP) is a collective term for diseases characterised by inflammation of the interstitium (e.g., the alveolar wall), causing restrictive lung disease. There are various causes for IP, such as drugs, collagen disease, viral infection, and radiation therapy^1^. In particular, idiopathic pulmonary fibrosis (IPF) has an average survival rate of 3–5 years and a poor prognosis^2^. In addition, acute exacerbation of IP generally has a very poor prognosis. However, IP treatments are currently limited to steroids, immunosuppressants, and antifibrotic drugs^3^, all of which have many side effects, such as dyspepsia, nausea, and photosensitivity; therefore, the development of new therapeutic agents to replace the current drugs is required.

Oxidative stress is one of the causes of exacerbation of symptoms, such as pulmonary fibrosis, in IP^4–6^. Glutathione (GSH) is an important antioxidant in the lungs, but the amount of reduced GSH in the epithelial lining fluid of patients with IPF is significantly reduced^7–10^. Moreover, patients' lung inflammatory cells produce high levels of oxides. High levels of 8-isoprostan, an oxidative stress marker, were also detected in the pulmonary bronchial lavage fluid^11^. Thus, it was observed that patients with IPF had an increase in oxides and a decrease in in vivo antioxidants, that is, a state of severe oxidative stress. The symptoms of bleomycin-induced IP mouse models deficient in Nuclear factor-erythroid 2-related factor 2 (NRF2: in vivo antioxidant system regulatory transcription factor)^12,13^ and in vivo antioxidant enzymes, such as Extracellular superoxide dismutase (EC-SOD)^6,14^ and catalase^15^, were worse than those of the wild-type IP mouse models. On the other hand, EC-SOD overexpression^15^, recombinant Manganese superoxide dismutase (MnSOD) administration^16^, and increased GSH levels by *N*-acetylcysteine administration^17^ alleviated the symptoms of bleomycin-induced IP mouse models. Moreover, administration of antioxidant polyphenols, such as mangoerin^18^, quercetin^19^, and resveratrol^20,21^, also mitigated the symptoms of bleomycin-induced IP mouse models. Thus, enhancement of in vivo antioxidant enzymes and external administration of antioxidants are very effective in alleviating the symptoms of IP. Antioxidants are thought to be effective therapeutic agents for IP.

Our silicon (Si)-based agents can react with water to produce hydrogen^22^. In particular, more hydrogen is generated in a weakly basic solution. Hydrogen is an excellent antioxidant that can specifically eliminate extremely toxic hydroxyl radical, which possesses the highest oxidation power among reactive oxygen species (ROS)^23^. Therefore, oral administration of a Si-based agent enables the continuous generation of a large amount of hydrogen in the body and is considered to be effective for diseases associated with oxidative stress. Our previous study demonstrated that Si-based agents generated hydrogen in intestinal tract and alleviated the symptoms of ulcerative colitis, Parkinson's disease, renal failure, skin flap ischemia–reperfusion injury and miscarriage caused by mother-to-child transmission^24–27^. Therefore, we hypothesised that Si-based agents might prevent fibrosis of the lung interstitium by reducing the oxidative stress and examined their effectiveness in IP, using drug-induced IP mouse models^28^.

## Results

**Figure 1:**
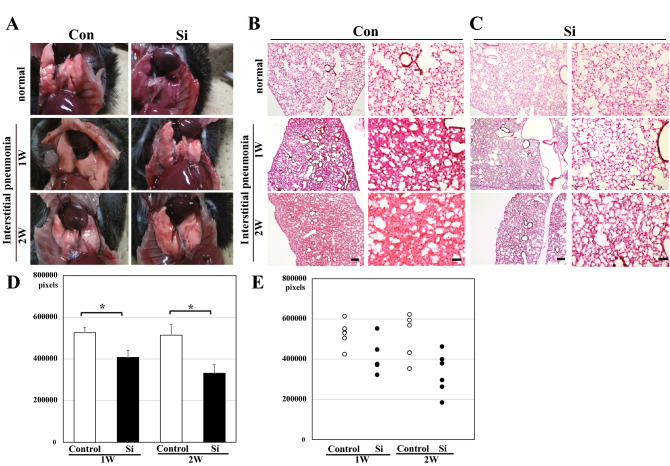
Gross pathology of the lung (**A**) and pathologic analysis using HE-stained lung specimens (**B**–**E**). (**A**) The representative photographs of the lung in the normal mice (upper panel) and IP mouse models treated with MTX for 1 week (middle panel) and 2 weeks (bottom panel). Left panel: Con group or Con-IP group; right panel: Si group or Si-IP group. (**B**,**C**) The representative microphotographs of the HE-stained lung in the normal mice (upper panel) and IP mouse models treated with MTX for 1 week (middle panel) and 2 weeks (bottom panel). (**B**) Con group or Con-IP group (**C**) Si group or Si-IP group. Left panel: low magnification; right panel: high magnification. Scale bar 200 µm (**B**,**C**: left); 100 µm (**B**,**C**: right). (**D**,**E**) The measured results of interstitial area in the lung. (**D**) Bar chart indicates the mean values. (**E**) The dot graph indicates the individual values. White: Control group. Black: Si-based agent group. MTX administration for 1 week (left pair graph) and 2 weeks (right pair graph). Data are expressed as mean. SEM of eight mice per group. **p < 0.01 vs. Control group, determined by Student’s paired *t*-test.

To examine whether the Si-based agent alleviated the symptoms of IP, we performed morphological analysis using the drug-induced IP mouse models treated with Methotrexate (MTX). The MTX-induced interstitial pneumonia model is widely used because lung fibrosis is clearly observed^29–31^. Control diet and Si-based agent diet were administered 1 week before MTX administration. Mice were randomly divided into the following four groups: Con group, control diet-fed normal mice; Si group, Si-based agent diet-fed normal mice; Con-IP group, control diet-fed IP mouse models; Si-IP group, Si-based agent-fed IP mouse models. First, the pulmonary abnormalities (e.g., haemorrhage and congestion) of the mice treated with MTX for a week or 2 weeks were investigated after thoracotomy under anaesthesia. As a result, there was no difference in macroscopic findings between normal and IP mouse models regardless of the type of diet or the administration period of MTX (Fig. 1A). Second, pathologic analyses using HE-stained lung samples were performed to examine the degree of haemorrhage, inflammation, and fibrogenesis that accompanied with IP. In the Con and Si groups, a spongiform appearance of the lungs contained many small cavities (pulmonary alveoli), some blood vessels, and a few bronchi (Fig. 1B). However, the lungs of the Con-IP group showed marked interstitial lung hypertrophy (Fig. 1B, Supplementary Fig. S1A). The degree of interstitial hypertrophy was greater at 2 weeks than at 1 week. In contrast, in the Si-IP group, a few mice models displayed moderate interstitial hypertrophy, but no interstitial hypertrophy was observed in majority of them, regardless of the administration period of MTX (Fig. 1C, Supplementary Fig. S1B). A comparative analysis of the lung interstitial area between the Con-IP and Si-IP groups revealed that interstitial enlargement was significantly suppressed in the Si-IP group compared to that in the Con-IP group (Fig. 1D, 1E).

**Figure 2:**
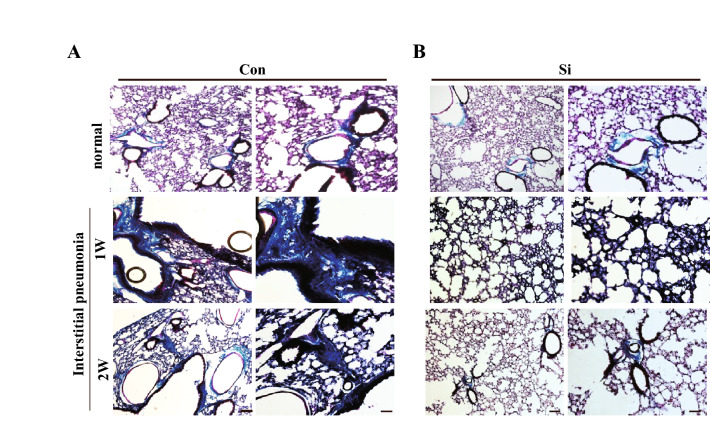
Pathologic analysis using Masson’s Trichrome-stained lung specimens. (**A**,**B**) The representative microphotographs of the lung in the normal mice (upper panel) and IP mouse models treated with MTX for 1 week (middle panel) and 2 weeks (bottom panel). (**A**) Con group or Con-IP group (**B**) Si group or Si-IP group. Scale bar 100 µm (**A**,**B**: left); 50 µm (**A**,**B**: right).

Subsequently, To further confirm that the increasing eosin-positive region observed by HE staining analysis was the lung interstitium., Masson's trichrome staining was performed on the state of collagen fibres that were greatly involved in early interstitial hypertrophy. Collagen fibres visualised in blue were observed only around the bronchi in both the Con and Si groups. However, in the Con-IP group, collagen fibres were observed not only around the bronchi but also in the interstitium (Fig. 2A). There was no significant difference between Masson's trichrome-stained images at 1 week and 2 weeks of MTX administration. In contrast, in the Si-IP group, most of the collagen fibres were observed only around the bronchi, regardless of the administration period of MTX (Fig. 2B).

**Figure 3:**
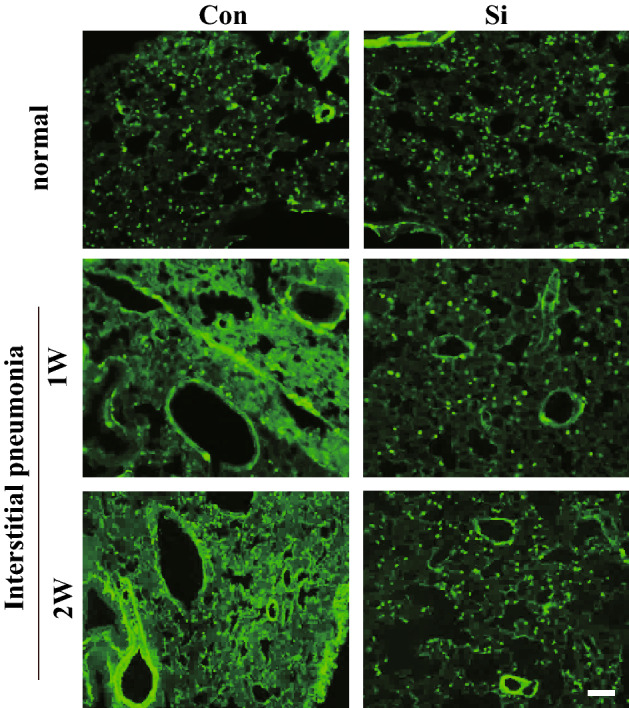
Immunofluorescence staining using α-SMA. The representative microphotographs of the lung in the normal mice (upper panel) and IP mouse models treated with MTX for 1 week (middle panel) and 2 weeks (bottom panel). Left panel: Con group or Con-IP group; right panel: Si group or Si-IP group. Scale bar: 20 µm.

As the symptoms of IP progress, smooth muscle actin filaments accumulate in the lung interstitium. To investigate the severity of IP, we performed fluorescent immunostaining for α-SMA. In the lungs of both Con and Si groups, α-SMA derived from smooth muscle cells in the lamina propria of the alveolar duct were detected in the dots (Fig. 3). However, in the Con-IP group treated with MTX for 1 week, it was found that most of the enlarged interstitium was stained uniformly, and similar morphology was observed for 2-week treatment as well. On the other hand, no such stained image was observed in the Si-IP group, and a dot-shaped stained image was observed in the lungs of the Si group regardless of MTX administration. Taken together, it was revealed that the Si-based agent alleviated interstitial hypertrophy which is the main lesion of IP.

**Figure 4:**
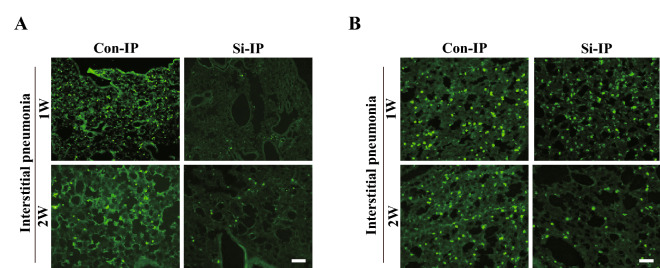
Immunofluorescence staining for Gr-1 (**A**) and F4/80 (**B**). The representative microphotographs of the lung in the IP mouse models treated with MTX for 1 week (upper panel) and 2 weeks (bottom panel). Left panel: Con-IP group; right panel: Si-IP group. Scale bar 20 µm.

Since the infiltration of immune cells is significantly involved in the fibrosis of interstitial pneumonia, we investigated the dynamics of neutrophils and macrophages in the lung interstitium using immunostaining for each immune cell marker. First, immunostaining for Gr-1, a neutrophil marker, was performed. In the Con-IP group, only a few positive cells were observed after 1 week of MTX administration, but many positive cells were observed after 2 weeks of its administration. However, such signals were hardly observed in the Si group treated with MTX for 1 week and only slightly for 2 weeks (Fig. 4A). Next, we conducted immunostaining using the F4/80 antibody, a macrophage marker. In the Con-IP group, infiltration of macrophages into the lung interstitium were observed regardless of the period of MTX administration. On the other hand, in the Si-IP group of both MTX administration periods, the number of macrophage cells infiltrating the lung interstitium was significantly reduced compared with that in the Con-IP group (Fig. 4B). Taken together, it was clarified that the infiltration of immune cells into the lung interstitium in IP was suppressed by the administration of the Si-based agent.

Finally, to investigate whether the oxidative stress associated with IP is alleviated by the administration of Si preparations, the Diacron-reactive oxygen metabolites (dROMs) and the Biological Antioxidant Potential (BAP) test were conducted using the IP mouse models treated with MTX for 2 weeks. The values of dROMs and BAP reflected blood oxidative metabolites and antioxidant capacity, respectively. In the dROM test, there were no difference was observed between the two groups, but the BAP value was significantly higher in the Si-IP group than in the Con-IP group. Moreover, when the ratio of BAP/dROMs was also examined as an index of oxidative stress, it was found that the Si-IP group was significantly higher than the Con-IP group, indicating that oxidative stress was reduced in the Si-IP group. Taken together, Si-based agent alleviated the oxidative stress associated with IP.

## Discussion

Morphological analyses revealed that the pulmonary interstitial hypertrophy of the Si-IP group was significantly reduced compared to that of the Con-IP group. In contrast, fluorescent immunostaining using immune cell marker antibodies clarified that the accumulation of immune cells was suppressed by the administration of the Si-based agent (Table 1). Furthermore, Si-based agent alleviated oxidative stress in the IP mouse models treated with MTX for 2 weeks. In conclusion, we demonstrated that the Si-based agent alleviated lung interstitial hypertrophy by suppressing immune cell accumulation induced by MTX.

**Table 1:** Analytical comparison results of Con-IP and Si-IP group.

Method	Target	1 week model	2 weeks model
Macroscopic findings	Pulmonary abnormalities (e.g., haemorrhage and congestion)	No difference	
HE staining	Interstitial hypertrophy	Control > Si = normal	Control > Si = normal
Masson’s trichrome staining	Collagen fibers in interstitium	Control > Si = normal	Control > Si = normal
Immunofluorescent staining	α-SMA expression in interstitium (α-SMA)	Control > Si	Control > Si
	The number of neutrophils (Gr-1)	Control > Si	Control > Si
	The number of macrophage cells (F4/80)	Control > Si	Control > Si

**Figure 5:**
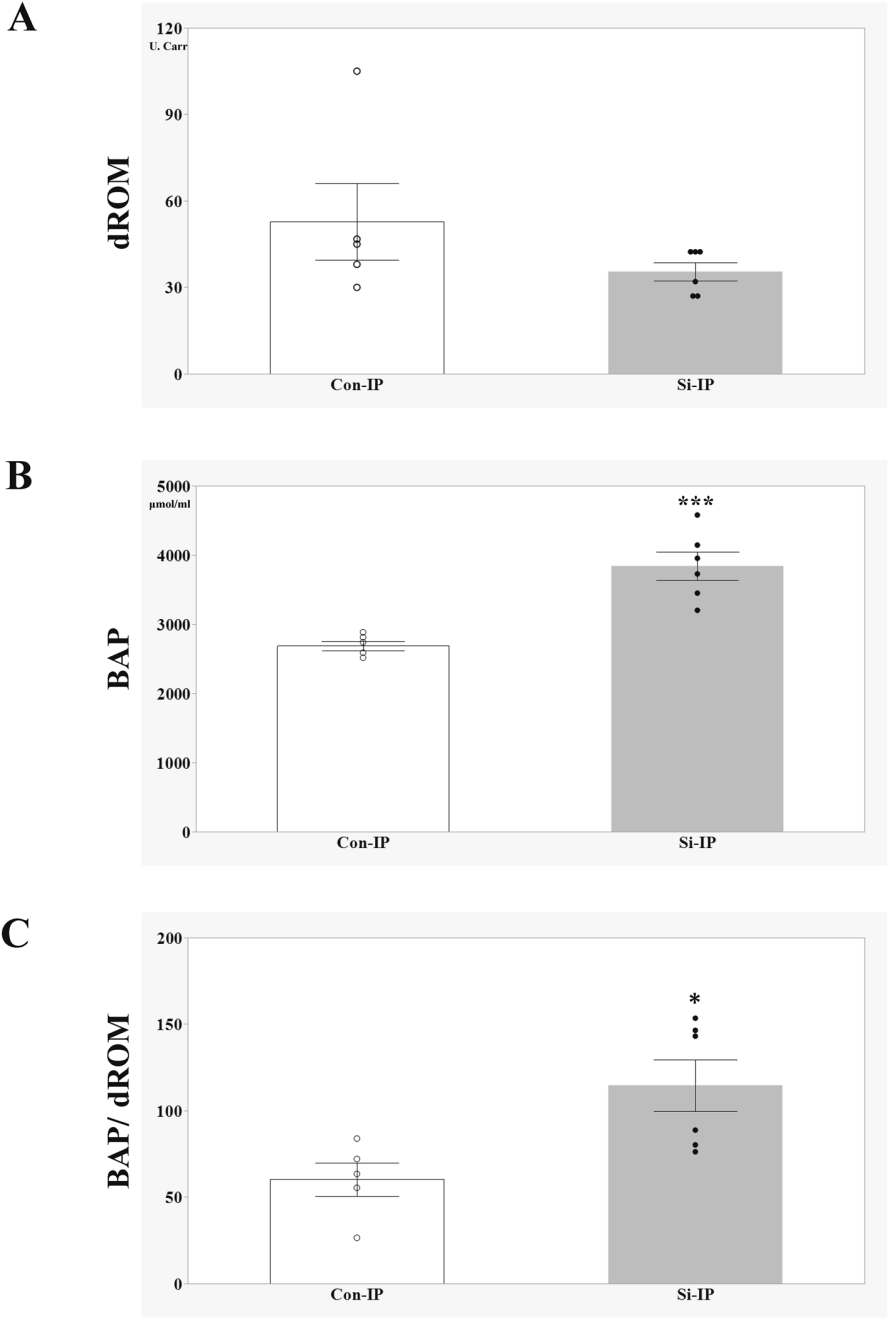
Evaluation of Oxidative stress in the IP mouse models treated with MTX for 2 weeks (**A**–**C**). The bar graph of the average of d-ROMs value (**A**), BAP value (**B**) and BAP/d-ROMs ratio (**C**). Con-IP (white, n = 5) and Si-IP group (grey, n = 6). Data are expressed as the mean ± standard error of the mean of five to six mice per group. *p < 0.05, ***p < 0.001 vs. Con-IP group, determined by Student’s t-test.

One of the hypotheses for the onset of MTX-induced IP is lung damage caused by an allergic response to MTX^28^. Infiltration of immune cells activated by MTX into the lungs and unabated continuation of the repair process after elimination of the inflammatory response leads to interstitial fibrosis^6^. In particular, transforming growth factor-β1 (TGF-β1) produced by macrophages is an inflammatory growth factor β1 with a strong chemotactic effect on inflammatory cells, and promotes inflammation to damage the lungs^6,32^. In addition, TGF-β1 activates nicotinamide adenine dinucleotide phosphate oxidase in myofibroblasts and promotes ROS production^33,34^. The release of ROS increases the release of TGF-β1 from lung epithelial cells through direct TGF-β1 activation^34,35^. TGF-β1 downregulates glutamate-cysteine ligase mRNA synthesis, the rate-limiting enzyme in the production of the antioxidant molecule GSH^36^. GSH synthesis is reduced in TGF-β1-overexpressing mice^37^. By reducing GSH, an important antioxidant in the lungs, ROS is further increased, which in turn increases TGF-β1 expression. Originally, TGF-β1 functioned to reduce inflammation and initiate repair^6^. TGF-β1 can also stimulate extracellular matrix accumulation by increasing the transcription of collagen mRNA^38^. However, persistent immune cell infiltration and oxidative stress induced by MTX administration promote the abnormal activity of TGF-β1, resulting in the acceleration of the lung fibrotic response^39^. Therefore, infiltration of immune cells into the lung is a hallmark of lung fibrosis. The Con-IP group showed infiltration of many immune cells into the lung, as well as significant lung fibrosis containing collagen fibres and myofibroblasts (Fig. 4). On the other hand, in the Si-IP group, only a small number of immune cells infiltrated into the lung and lung fibrosis was hardly observed. Moreover, Si-based agent significantly alleviated oxidative stress associated with IP (Fig. 5). Taken together, it is considered that the elimination of ROS by the antioxidant action of the Si-based agent led to the break-down of a vicious cycle of increased oxidative stress and abnormal activity of TGF-β, and hence alleviated lung fibrosis.

The lung which is the site of respiration and energy metabolism is constantly exposed to extremely high oxygen partial pressure and is always susceptible to oxidative stress. In animal experiments, deletion of in vivo antioxidant-related substances (NRF2, EC-SOD, and catarase) has been shown to exacerbate the symptoms of interstitial pneumonia^6,11,13,14,16^ and excessive ROS is significantly involved in lung fibrosis. The matrix metalloproteinase (MMP) family, mainly MMP7 is involved in lung fibrosis. MMP is overexpressed in the lungs of patients with IPF^40,41^. ROS inactivate MMP inhibitors and promote MMP activation by directly inducing transcription^41,42^. For example, hypochlorous acid activates MMP7^43^, and hydrogen peroxide and peroxynitrite activates both MMP2 and MMP9^42^. Thus, ROS exacerbates the symptoms of IP by promoting lung damage and MMP-mediated fibrosis. Therefore, it is important to eliminate oxidative stress by administering antioxidants. The administration of some polyphenols was effective in alleviating the symptoms of IP^19–22^. However, since polyphenols eliminate all active oxygen, there is a risk of reducing the bactericidal action of the lungs. On the other hand, hydrogen is an antioxidant that can overcome such disadvantages. Since hydrogen specifically eliminates harmful ROS (e.g., hydroxy radicals) and has no side effects, it is effective in alleviating diseases associated with oxidative stress (e.g., rheumatoid arthritis-associated IP)^43^. However, it is difficult to use the conventional hydrogen administration method because it is difficult to maintain the hydrogen concentration in hydrogen-rich water, and there is a risk of explosion of hydrogen gas. Our Si-based agent can react with water to generate large amounts of hydrogen continuously. Therefore, Si-based agents can be a breakthrough for the in vivo administration of hydrogen. Oral administration of Si-based agents alleviated the symptoms of ulcerative colitis, Parkinson's disease, renal failure, skin flap ischemia–reperfusion injury and miscarriage caused by mother-to-child transmission^24–27^. Taken together, since Si-based agents can sufficiently supply hydrogen and have no side effects in the living body, they are considered to be effective for the treatment of IP.

Since 2019, many people have died of IP associated with COVID-19^44^. Surprisingly, inhalation of high concentrations of hydrogen gas alleviated the symptoms of IP^45^. Although hydrogen has been proven to be effective in patients with coronavirus-induced IP, However, high-concentration hydrogen gas can only be used in specific facilities such as hospitals as there is a risk of explosion, and therefore requires careful handling. On the other hand, the administration of the Si-based agent is simple and safe and can be prescribed to home care recipients. It is also considered to be effective for other forms of IP, apart from drug-induced IP. In the near future, Si-based agents could be an effective treatment for all IPs, including IPF and COVID-19-induced IP.

## Materials and methods

### Methotrexate-induced interstitial pneumonia model preparation

Six-week-old C57Bl/6J male mice were purchased from Japan SLC (Shizuoka, Japan). The mice were kept at 23–25 °C and fed both custom-ordered rodent chow and water ad libitum. Mice were randomly divided into the following 6 groups (6 mice/group): Con group, control diet-fed normal mice; Si group, Si-based agent diet-fed normal mice; Con-IP group (1 or 2 week), control diet-fed IP mouse models; Si-IP group (1 or 2 week), Si-based agent-fed IP mouse models. Methotrexate (MTX)-induced mouse models were prepared as previous described^29–31^. MTX (FUJIFILM Wako Chemicals Corporation, Osaka, Japan) was suspended in 50 mM Na_2_CO_3_, added to a 10 mg/mL solution. IP was induced by daily oral administration (3 µg/g weight) of MTX using a feeding needle (Thermo Fisher Scientific, Waltham, MA), as indicated in Table 2. According to clinical guidelines^46^, a 2-day washout period was set after 5 days of MTX administration. We did one preliminary test and two main tests (3 mice/group/test). In mouse rearing, since 3 mice/cage were used, this test was divided into 2 times, resulting in 6 mice per group. Including the preliminary test, the total number of mice used in the experiment is 54. Six mice per group in main tests were used for analysis. Mice that died during modelling were exclude from analysis.

**Table 2:**
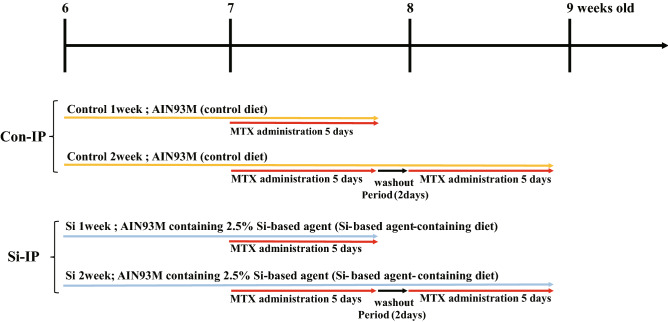
Experimental design.

All animal research was approved by the animal ethics committee of Osaka University (approval number 02-001-003) according to the National Institute of Health Guide for Care and Use of Laboratory Animals. This study was conducted in compliance with ARRIVE guidelines. Every effort was made to minimise the number and suffering of the experimental animals. After the experimental treatment, animals are carefully observed and analgesic nonsteroidal anti-inflammatory agent (NSAID: ibuprofen; 5 mg/kg) is administered intraperitoneally when pain-indicating behaviour is observed. When it is judged that normal feeding and water supply are difficult, food is placed on the bedding, and supplemental agar jelly is used. In addition, if abnormalities are observed in experimental animals, or if hypothetical humane endpoints (e.g., difficulty in feeding/watering, breathing problems, self-harm, rapid weight loss of 20% or more in a few days) are observed, the target animals were immediately euthanized by intraperitoneal administration of pentobarbital (200 mg/kg).

### Si-based agent and diets

Si-based agent were prepared as previous described^22,25^. Si-based agent was fabricated from intrinsic (i-type) polycrystalline Si. After pulverization using the bead milling method, the surface treatment was performed to improve the hydrogen generation ability.

In addition, two customised rodent diets (Oriental Yeast Co., Ltd., Tokyo, Japan), AIN93M (control diet), and AIN93M containing 2.5% Si-based agent (Si-based agent-containing diet) were prepared as previously described^25^. Mice were fed each diet 1 week before MTX administration, taking into account individual differences in food intake.

### Frozen sample preparation

Under deep anaesthesia with a combination anesthetic (0.3 mg/kg medetomidine, 4.0 mg/kg midazolam, and 5.0 mg/kg butorphanol)^47^, mice were perfused with 4% paraformaldehyde in 0.1 M phosphate buffer (PB: pH 7.4). After fixation with the same fixative, the removed lungs were cryoprotected in a 30% sucrose solution and then frozen with dry ice. The frozen samples were sectioned into 20-µm thick slices, mounted in MAS-coated glass slides (Matsunami-glass, Osaka, Japan), and stored at − 80 °C until use.

### Tissue staining

Lung samples were air-dried for an hour before staining. For haematoxylin and eosin (HE) staining, the samples were stained with haematoxylin solution (FUJIFILM Wako Chemicals Corporation, Osaka, Japan) for 5 min, washed with running water for 10 min, and then stained with eosin solution (FUJIFILM Wako Chemicals Corporation) for 3 min. For Masson’s trichrome staining, the samples were stained using Masson’s Trichrome staining solution kit (Muto Pure Chemicals Co., Ltd., Tokyo, Japan) according to the manufacturer’s instructions. Collagen fibres were stained blue with aniline blue, nuclei were stained dark purple with Weigert’s iron haematoxylin solution, and cytoplasm was stained red with fuchsin acid. The reaction times were as follows: aniline blue (2.5 min), Weigert’s iron haematoxylin solution (10 min), and fuchsin acid (25 min). After ethanol dehydration and xylene immersion, both stained samples were sealed with cover glasses using Entellan New (Merck Millipore, Burlington, MA). All slides were analysed under a BX53 microscope (Olympus Corporation, Tokyo, Japan).

### Antibodies

The primary antibodies used for immunofluorescence staining were rabbit anti-α-smooth muscle actin filament (α-SMA) polyclonal antibody (1:100; catalogue no. M0851, Dako, Bath, UK), rat anti-Gr-1 monoclonal antibody (1:1000; catalogue no. MAB1037, R&D systems, Minneapolis, MN), and rat anti-F4/80 monoclonal antibody (1:100; catalogue no. ab6640, Abcam, Cambridge, UK). The secondary antibodies (1:500, Thermo Fisher Scientific) used for immunofluorescence staining were donkey anti-mouse IgG polyclonal antibody conjugated with Alexa Fluor 488 (catalogue no. A-21202), and goat anti-rat IgG polyclonal antibody conjugated with Alexa Fluor 488 (catalogue no. A-11006).

### Immunofluorescence staining

After rinsing with 0.01 M phosphate-buffered saline (PBS), the samples were treated with the blocking solution (0.3% Triton-X, 3% bovine serum albumin in 0.01 M PBS) for 30 min and then incubated with the primary antibody in blocking buffer at 4 °C overnight. After washing several times, the slides were incubated with the secondary antibody corresponding to each primary antibody for 1 h. The wash in 0.01 M PBS was followed by mounting using PermaFluor (Thermo Fisher Scientific). All slides were analysed under a BX53 microscope (Olympus Corporation).

### Oxidative stress measurement

Oxidative stress measurement was performed as previously described^24^. Under deep anesthesia, whole blood was acquired from the right atrium of the following groups: 2-week-IP-Con and 2-week-IP-Si groups. Blood was centrifuged (3000 rpm, 10 min, 4 °C) and serum was gathered. The serum was stocked at − 80 °C until use. To examine the serum levels of ROS metabolites and anti-oxidative capacity, the levels of ROS metabolite-derived compounds (dROMs) and biological antioxidant potential (BAP) were measured by REDOXLIBLA (Wismerll Co. Ltd., Tokyo, Japan). The results of dROM test were shown as arbitrary units (U. Carr); 1 U Carr corresponds to 0.8 mg/L of hydrogen peroxide^48^. BAP indicated the reducing power of blood using the amount of trivalent iron ions (µM) reduced to divalent iron ions as an indicator. Comparative analysis was performed using the dROMs value, BAP value and the ratio of BAP divided by dROMs.

### Image analyses

For HE staining, four micrographs of lung tissue per individual were analysed using ImageJ software (1.52a version; National Institute of Health, USA) to quantify the area of the lung interstitium. The average value of the four photographs was taken as the interstitial area of each lung. Student’s *t*-test was conducted to compare the differences between the Si-based agent-treated group and non-treated group. The results of the statistical analyses were expressed as mean values ± standard error of the mean (SEM) and compared with the non-treated group. *p value < 0.05 was considered significant.

## Supplementary Information

Supplementary Information.

## Data Availability

All relevant data are within the paper and its Supplementary Information files.
