# Supplementary material for: Effect of the new silicon-based agent on the symptoms of interstitial pneumonitis
Source: Sci Rep. 2023 Apr 7;13:5707. doi: 10.1038/s41598-023-32745-8 (PMC10080516; doi:10.1038/s41598-023-32745-8)
Supplement: Supplementary file 1 — Supplementary Information. [file 41598_2023_32745_MOESM1_ESM.pdf]

Supplemental Fig. 1

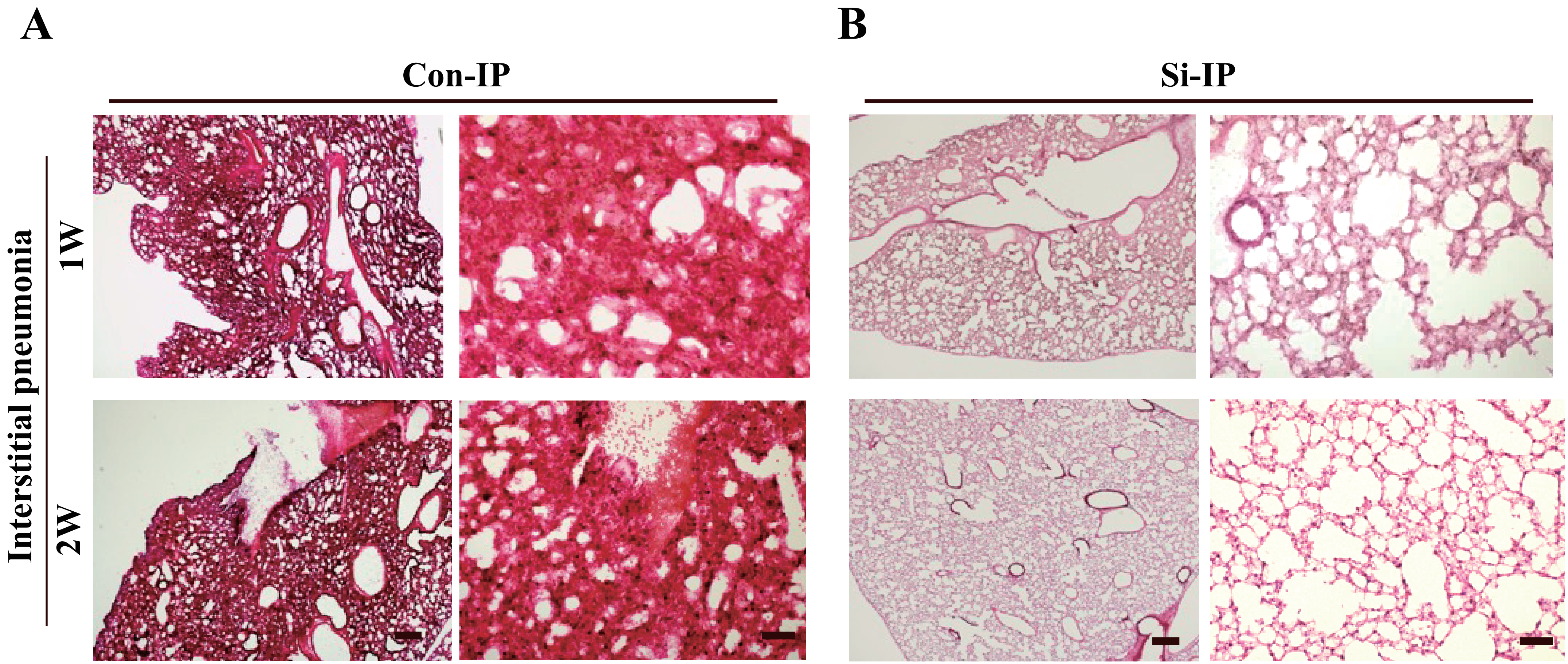

Supplementary Fig. S1

Pathologic analysis of HE-stained lung specimens (A, B).

Additional microphotographs of the lung in the IP model mice treated with MTX for 1 week (upper panel) and 2 weeks (bottom panel). (A) Con-IP group (B) Si-IP group. Scale bar: 200 µm (A, B: left); 100 µm (A, B: right).
